# Teacher unfairness in adolescence, educational attainment, and adult Health: The role of school- and individual-level perceptions tested in a national cohort study

**DOI:** 10.1016/j.ssmph.2025.101810

**Published:** 2025-04-25

**Authors:** Shanting Chen, Stephanie Koning, Jessica Polos, Phoebe Lam, Taylor Hargrove, Natalie Ebner, Jacob Aronoff, Thomas McDade

**Affiliations:** a University of Florida, United States; b University of Nevada, Reno, United States; c DePaul University, United States; d Carnegie Mellon University, United States; e University of North Carolina at Chapel Hill, United States; f Arizona State University, United States; g Northwestern University, United States

## Abstract

• Teacher unfairness in adolescence predicted higher depression.
• Greater perceived teacher unfairness relative to peers predicted higher depression.
• School-wide teacher unfairness predicted poorer self-rated health in adulthood.
• Educational attainment mediates the link of teacher unfairness and adult health.

## 1 Introduction

Adolescents spend a significant portion of their lives in school (Huang, 2020), where the social climate plays a critical role in shaping their development. Teacher unfairness, a key contributor to a negative school climate, can undermine students' motivation and engagement, leading to lower academic achievement and educational attainment (Helm et al., 2020; Ripski & Gregory, 2009). Educational attainment, in turn, is a well-documented determinant of health, associated with a wide range of physical and mental health outcomes, including disparities in population-level disease burden, morbidity, and mortality (Masters et al., 2015). Thus, understanding whether educational attainment serves as a mechanism linking teacher unfairness to subsequent health is crucial to inform interventions aimed at reducing health disparities and improving population health.

### 1.1 The life-course effects of teacher unfair treatment and health outcomes

Student perceptions of teacher unfairness (Gini et al., 2018) represent a critical but underexplored dimension of school climate, which functions as a significant social determinant of health (Viner et al., 2012). Adolescence is a particularly vulnerable developmental period during which negative experiences, such as perceived teacher unfairness, can have profound and lasting impacts. During adolescence, heightened sensitivity to social evaluation and authority figures (Spencer, 2007; Sumter et al., 2010) makes exposure to unfair treatment particularly impactful. Such experiences can trigger chronic stress and psychosocial distress, potentially increasing the risk for long-term health. Indeed, prior research has linked teacher unfairness to adverse adolescent health outcomes, including somatic complaints like headaches (Lenzi et al., 2013; Santinello et al., 2009), psychological distress such as depression and anxiety (Gini et al., 2018; Roeser et al., 1998), and poor academic outcomes—such as reduced school belonging, motivation, engagement, and satisfaction (F. Chen & Cui, 2020; Griffith et al., 2022; Helm et al., 2020; Huang, 2020; Ripski & Gregory, 2009).

However, a limitation of previous empirical research is that it has primarily relied on cross-sectional data, leaving the long-term impact of perceived teacher unfair treatment largely unexplored. According to the life course health development framework (Halfon & Hochstein, 2002), social inequities during adolescence can shape biological, psychological, and social trajectories, leading to cumulative health risks across the lifespan. This study investigates how perceptions of teacher unfairness during adolescence influence mental health, with a specific focus on depressive symptoms. Beyond mental health, we also explore the physical health consequences of such unfair treatment. Teacher unfairness can disrupt the stress response system, and chronic exposure to stress may increase the risk of developing chronic diseases (Goosby et al., 2017). To provide a comprehensive understanding of physical health, we examine both self-reported health and an objective biomarker of metabolic syndrome, a key biological indicator linked to increased risk of chronic diseases such as heart attack and diabetes (Mottillo et al., 2010). We focus specifically on health outcomes in young adulthood, a developmental stage that plays a pivotal role in shaping long-term life trajectories through key milestones such as educational attainment and entry into the workforce (Wood et al., 2018).

### 1.2 Perceptions of teacher unfairness at the individual, school-wide, and individual-school discrepancy levels

While perceived teacher unfairness has often been studied as an individual-level risk factor, school-wide norms play a critical role in shaping student behavior and experiences (Benner et al., 2022; Kumar et al., 2002). School-level perceptions of teacher unfairness provide valuable insight into the broader social context that influences health and development (Bronfenbrenner & Morris, 2006), which has been largely overlooked in prior research. Moreover, alignment, or lack thereof, between individual and school-wide perceptions of teacher unfairness provides valuable insight into a student's experience. A greater mismatch, where a student's perception diverges from that of their peers, may indicate that unfairness is a marginalizing experience, as adolescents frequently use their peers as a reference point for self-evaluation, shaping their self-worth and decision-making (Ciranka & van den Bos, 2019; Crosnoe, 2009). This individual-school match, often described by concepts like the “*looking glass self*” or the “*frog pond effect*” (Cooley, 1902; Marsh & Hau, 2003), suggests that fitting in with peers fosters social integration and minimizes maladjustment, while a mismatch can contribute to stress and poor outcomes (Anderman, 2002; Benner et al., 2015). As such, understanding both individual and school-wide perceptions of unfairness is critical for capturing the full impact on student well-being.

### 1.3 Mediating role of educational attainment

Educational attainment is a crucial social determinant of health (Viner et al., 2012), shaping access to resources and opportunities that protect and promote well-being (Phelan & Link, 2005). It significantly impacts a wide range of mental and physical health conditions, including depression, heart disease, and diabetes (Cutler & Lleras-Muney, 2006; Miech & Shanahan, 2000; Noppert et al., 2021). As a result, education policy is widely recognized as a crucial strategy for improving population health and reducing health disparities (Galea et al., 2011; Woolf et al., 2007).

Perceived teacher unfairness— a modifiable aspect of the school climate and a critical target for intervention—can undermine educational attainment by reducing students’ engagement, motivation, and grades (Helm et al., 2020; Ripski & Gregory, 2009). These negative academic outcomes can contribute to higher dropout rates and lower educational attainment overall (Lee & Breen, 2007), further limiting access to essential resources (e.g., fulfilling jobs, economic security, and healthy lifestyles; Mirowsky & Ross, 2003), which in turn, affect long-term mental and physical health. Despite these broad and enduring impacts, the long-term impact of perceived unfair teacher treatment during adolescence on adult health, particularly through its effect on educational attainment, remains understudied. This research gap is what the current study seeks to address.

### 1.4 Current study

This study has two primary aims. First, it investigates the long-term effects of perceived teacher unfairness during adolescence—examined at the individual level, school level, and discrepancies between the two—on adult health outcomes, including depression, self-reported health, and metabolic syndrome. We hypothesize that greater perceived teacher unfairness, either individually or school-wide, will predict worse health outcomes (i.e., higher depression, worse self-reported health, and higher metabolic syndrome), with more pronounced effects when individuals perceive higher unfairness compared to their peers. Second, the study examines whether educational attainment mediates these links, hypothesizing that higher perceived unfairness leads to lower educational attainment, which in turn negatively impacts adult health.

## 2 Methods

### 2.1 Participants

Data were drawn from Waves 1 and 4 of the National Longitudinal Study of Adolescent to Adult Health (Add Health), a nationally representative cohort of adolescents in grades 7 through 12 in the United States in 1994–1995. At Wave I, stage 1 involved the recruitment of a stratified random sample of all high schools in the U.S., along with a feeder middle school for each high school (Harris et al., 2019). In total, 144 schools were enrolled, and in-school questionnaires were administered to 90,118 adolescents (all adolescents at each school; density = 25 -2559 students). Stage 2 of Wave I (1994–1995) involved recruiting an in-home sample of 20,745 adolescents ages 11–20, based on student enrollment rosters from participating schools. At Wave 4 (2008) 14,800 participants (71 % of the original in-home sample), ages 24–32 years, participated in another in-home data collection. At Wave I, school administrators of every participating school were sent a questionnaire. Data sources included the in-school survey, in-home interviews, and school administrator questions at Wave I, as well as in-home interviews and biological data collected at Wave IV. Add Health was approved by the institutional review board of the University of North Carolina, Chapel Hill, and all participants provided written informed consent.

Our analytic sample included individuals who participated in both Wave I and Wave IV of Add Health (n = 15,701). Exclusions were made for individuals with missing survey weights (*n* = 901; 8 % of the final sample). As the focus of this study was on the school experience of adolescents, at the school level, we excluded schools that had very few numbers of students (less than 20 followed by Chen et al., 2023). This reduced the total number of schools from 144 to 130. Additionally, individuals who reported being pregnant or probably pregnant at Wave IV (*n* = 487; 3 % of the final sample) were excluded due to the significant variations in mental and physiological health during pregnancy compared to non-pregnant periods. With these restrictions in place, our final analytical sample size was 14,245 from 130 schools. The sample was 49 % male and racially/ethnically diverse (53 % White, 20 % Black, 16 % Latinx, 6 % Asian, and 5 % other race/ethnicity).

### 2.2 Measures

Table 1 provides basic demographic characteristics for the individuals included in the final analytic sample and their schools. Table 2, Table 3 display the correlations of demographic and study variables at the individual- and school-level for the final analytic sample.

**Table 1:** Characteristics of adolescents and their schools.

	N	Frequency (%)	M (SD)	Skewness	Kurtosis
**Primary construct of interest**					
Student Perceived Teacher Unfair Treatment	9917		2.60 (1.12)	0.47	−0.40
School-wide Perceived Teacher Unfair Treatment	130		2.58 (0.27)	−0.18	1.41
Individual-School Discrepancy Scores	9917		0.00 (1.1)	0.46	−0.34
Educational Attainment (W4)	14244		5.68 (2.19)	0.35	0.76
Depressive Symptoms (W4)	14223		5.24 (4.11)	1.29	2.16
Self-Reported Health (W4)	14245		3.65 (0.92)	−0.26	−0.36
Metabolic Syndrome (W4)	11880		1.78 (1.23)	0.36	−0.52
**Adolescent covariates**					
Race					
White	7565	53			
Black	2882	20			
Latinx	2263	16			
Asian	815	5.9			
Other	708	5			
Male	6921	49			
Age (W1)	14235		16.13 (1.7)	−0.14	−0.86
Age (W4)	14235		29.1 (1.75)	−0.11	−0.78
Parent(S) with Postsecondary Degree	14245	47.4			
Depressive Symptoms (W1)	14195		5.92 (4.26)	1.00	1.13
Self-Reported Health (W1)	14233		3.88 (0.91)	−0.44	−0.45
BMI (W1)	13902		22.66 (4.53)	1.47	3.58
GPA (W1)	7095		2.88 (0.77)	−0.41	−0.61
Household Income (In Deciles)	10802		5.48 (2.85)	0.01	−1.23
**School covariates**					
Percent Minority Students	130	42			
Percent New Teacher	128	10			
Percent Teacher at School 5 Years Or More	122	66			
School Size	130				
Small (1–400 students)	29	22			
Median (401–1000 students)	61	47			
Large (1001–4000 students)	40	31			
Percent Of Parents with Postsecondary Degree	130	45			
Percent Of Family Received Welfare	130	11			

*Note.* W1 = Wave I, W4 = Wave IV. School characteristics are at the school level (*N* = 130). All other variables are at the student level (*N* = 14,245). ∗*p* < .05, ∗∗*p* < .01, ∗∗∗*p* < .001.

**Table 2:** Correlations for key study constructs and covariates at the individual level.

	1	2	3	4	5	6	7	8	9	10	11	12	13	14	15	16	17	18
1. Student Perceived Teacher Unfairness (W1)	–																	
2. Individual-School Discrepancy Scores (W1)	0.98∗∗	–																
3. Educational Attainment (W4)	−0.08∗∗	−0.07∗∗	–															
4. Depressive Symptoms (W4)	0.10∗∗	0.09∗∗	−0.17∗∗	–														
5. Self-Reported Health (W4)	−0.10∗∗	−0.08∗∗	0.21∗∗	−0.28∗∗	–													
6. Metabolic Syndrome (W4)	0.02	0.01	−0.15∗∗	0.04∗∗	−0.28∗∗	–												
7. Latinx	−0.01	−0.01	−0.08∗∗	0.01	−0.05∗∗	0.04∗∗	–											
8. Black	0.04∗∗	0.02	−0.04∗∗	0.05∗∗	−0.02∗	0.08∗∗	−0.22∗∗	–										
9. Asian	−0.03∗∗	−0.03∗	0.09∗∗	0.02∗	−0.02∗	−0.01	−0.11∗∗	−0.12∗∗	–									
10. Other	0.030∗∗	0.03∗∗	−0.01	0.02∗∗	−0.03∗∗	0.01	−0.10∗∗	−0.12∗∗	−0.06∗∗	–								
11. White	−0.028∗∗	−0.01	0.05∗∗	−0.07∗∗	0.07∗∗	−0.09∗∗	−0.46∗∗	−0.54∗∗	−0.26∗∗	−0.24∗∗	–							
12. Gender (Male = 1, female = 0)	−0.025∗	−0.02∗	−0.12∗∗	−0.10∗∗	0.05∗∗	0.15∗∗	0.01	−0.04∗∗	0.02∗∗	−0.01	0.02∗	–						
13. Parental Education	−0.045∗∗	−0.03∗∗	0.33∗∗	−0.09∗∗	0.13∗∗	−0.10∗∗	−0.17∗∗	0.01	0.07∗∗	0.02∗∗	0.07∗∗	0.02∗∗	–					
14. Age (W1)	0.081∗∗	0.04∗∗	−0.02∗	0.00	−0.02∗	0.08∗∗	0.08∗∗	−0.03∗∗	0.07∗∗	−0.03∗∗	−0.06∗∗	0.05∗∗	−0.03∗∗	–				
15. GPA (W1)	−0.18∗∗	−0.15∗∗	0.43∗∗	−0.14∗∗	0.15∗∗	−0.14∗∗	−0.11∗∗	−0.15∗∗	0.11∗∗	0.00	0.14∗∗	−0.10∗∗	0.19∗∗	−0.09∗∗	–			
16. Depressive Symptoms (W1)	0.17∗∗	0.16∗∗	−0.16∗∗	0.31∗∗	−0.17∗∗	0.04∗∗	0.07∗∗	0.03∗∗	0.06∗∗	0.01	−0.11∗∗	−0.16∗∗	−0.11∗∗	0.11∗∗	−0.18∗∗	–		
17. Self-Reported Health (W1)	−0.12∗∗	−0.12∗∗	0.16∗∗	−0.14∗∗	0.28∗∗	−0.10∗∗	−0.04∗∗	0.03∗∗	−0.02∗	−0.02∗	0.03∗∗	0.11∗∗	0.10∗∗	−0.02∗∗	0.14∗∗	−0.28∗∗	–	
18. BMI (W1)	0.03∗∗	0.02	−0.10∗∗	0.06∗∗	−0.23∗∗	0.40∗∗	0.06∗∗	0.07∗∗	−0.03∗∗	0.02	−0.09∗∗	0.04∗∗	−0.08∗∗	0.19∗∗	−0.13∗∗	0.07∗∗	−0.22∗∗	–
19. Household Income (W1)	−0.06∗∗	−0.05∗∗	0.34∗∗	−0.12∗∗	0.13∗∗	−0.11∗∗	−0.17∗∗	−0.19∗∗	0.06∗∗	0.00	0.24∗∗	0.02∗	0.37∗∗	−0.01	0.22∗∗	−0.11∗∗	0.10∗∗	−0.10∗∗

*Note.* W1 = Wave I, W4 = Wave IV. Parental education = Parent(s) with postsecondary degree. ∗p < .05, ∗∗p < .01, ∗∗∗p < .001.

**Table 3:** Correlations for key study constructs and covariates at the school level.

	1	2	3	4	5	6
1. School-wide Teacher Unfairness (W1)	–					
2. Percent of Minority Students (W1)	0.22∗	–				
3. School Size (W1)	0.26∗∗	0.20∗	–			
4. Percent of New Teachers (W1)	−0.03	0.01	0.02	–		
5. Percent of Teachers Who Worked at the Same School for Five Years or More (W1)	0.09	−0.14	0.21∗	−0.22∗	–	
6. Percent of Households Receiving Welfare (W1)	0.13	0.52∗∗	−0.06	−0.14	0.02	–
7. Percent of Parents with Postsecondary Degree (W1)	−0.14	−0.22∗	0.10	0.24∗∗	−0.03	−0.66∗∗

*Note.* W1 = Wave I. ∗p < .05, ∗∗p < .01, ∗∗∗p < .001.

*Perceptions of teacher unfair treatment at school.* In the Wave I in-school survey, adolescents rated their agreement with the statement: “Teachers at your school treat students fairly” on the original scale ranging from 1 (*strongly disagree*) to 5 (*strongly agree*). This item was then reverse coded so that higher score represented higher levels of unfair teacher treatment at school. We aggregated this item for all adolescents in each school to obtain a school-wide average.

Individual-school discrepancy scores were calculated by subtracting the school-wide teacher unfair treatment mean from each adolescent's individual report (*M* = −0.00, *SD* = 1.10). This approach has been used in previous studies of Add Health that capture individual-school discrepancy of prejudice and school belonging (Benner et al., 2015; E. Chen et al., 2023). These scores are continuous, with higher (positive) scores indicating that adolescents perceived more teacher unfairness than their schoolmates and lower (negative) scores indicating that schoolmates perceived more teacher unfair treatment than the adolescents. This discrepancy score essentially gauged the magnitude of divergence between adolescents and their schoolmates.

*Educational attainment.* At Wave IV, individuals were asked “What is the highest level of education that you have achieved to date?” in the In-Home Survey. There were thirteen response categories ranging from 1 (*8*th *grade or less*) to 13 (*completed post-baccalaureate professional education*) with 5 representing completed high school.

*Depressive symptoms.* Depressive symptoms were measured by the Center for Epidemiologic Studies Depression scale, and we used 9 items that were asked at both Wave I and Wave IV in the In-Home Survey (Desch et al., 2023). Respondents were asked how often in the past seven days they felt: “depressed,” “happy,” “bothered by things that don't usually bother you”, etc. Responses ranged from 1 (*never*) to 3 (*most or all the time*). Positively framed questions were reverse scored, and values were summed across questions, with possible composite scores of depressive symptoms ranging from 9 to 27 with higher scores indicating higher levels of depressive symptoms (α = .80 and 0.81 at Wave I and IV respectively for the 9-item scale)

*Self-reported health.* Participants provided a general rating of their physical health by responding to the question, “In general, how is your health?” at both Wave I and Wave IV in the In-Home Survey. Responses ranged from 1 (*excellent*) to 5 (*poor*). The scale was recoded so higher scores reflected better self-reported health.

*Metabolic syndrome.* At Wave IV, the diagnosis of metabolic syndrome was determined using the five criteria outlined by the International Diabetes Federation (IDF, Alberti et al., 2009). Sex differences in the computation of certain metabolic syndrome components are based on well-documented biological and physiological distinctions between men and women (Alberti et al., 2009; Grundy et al., 2004). First, participants were categorized as having *central adiposity* if their measured waist circumference was equal to or greater than 88 cm for females and 102 cm for males. Second, *elevated blood pressure* was defined based on one of the following criteria: self-report of antihypertensive medication use, diagnosis of hypertension by a medical professional, or measured resting values ≥ 130 mmHg systolic or ≥85 mmHg diastolic. Third, participants were considered to have *elevated triglycerides* if they fell within the top three deciles of the sample distribution for males and the top two deciles for females. Fourth, elevated glucose levels were determined based on glycated hemoglobin (HbA1c) levels ≥5.7 %, a threshold commonly used in epidemiologic cohorts (Ong et al., 2010) and consistent with previous Add Health analyses (Gaydosh et al., 2018; Miller et al., 2020). Fifth, *lowered High-density lipoprotein (HDL)* was defined for males within the bottom two deciles and females within the bottom three deciles of the sample distribution. Metabolic syndrome can manifest along a continuum of severity (Goodman, 2008). To quantify this, a metabolic syndrome count score was calculated for each participant, representing the number of criteria exceeding the IDF thresholds (ranging from 0 to 5). This definition of metabolic syndrome has been used in previous studies of Add Health participants (E. Chen et al., 2023).

*Individual- and school-level covariates.* For controls at the individual level, adolescents reported their biological sex (1 = male, 0 = female), age, race/ethnicity, parent education (0 = parents with no postsecondary degree, 1 = parents with postsecondary degree), and family household income (coded in deciles) at Wave I. For race/ethnicity, during Wave I, individuals were asked, “What is your race?” followed by a separate question: “Are you of Hispanic or Latino origin?” Respondents were classified as non-Hispanic White if they identified as White and did not report being of Hispanic or Latino origin. Those who answered "yes" to being of Hispanic or Latino origin were classified as Latino. Non-Hispanic White was designated as the reference group. Adolescents reported their grades in the four core content areas (English, mathematics, social studies, and science) in Wave I. Ratings ranged from 1 (*D/F*) to 4 (*A*) and were averaged across subjects and then converted to a standard 4-point composite grade point average (GPA) with a higher score indicating higher GPA. At the school level, we included measures of school size, percentage of new teachers, percentage of teachers who worked at the same school for five years or more, percentage of minority students (subtracting the percent of white students from 100 %), percentage of households receiving welfare, and percent of parents with postsecondary degree. These covariates were added because previous research has shown they are associated with mental and physical health outcomes in prior literature (e.g., Brännlund et al., 2017; E. Chen et al., 2006; Read & Gorman, 2006).

### 2.3 Analysis plan

All analyses were conducted in a multilevel structural equation modeling (MSEM) framework in *Mplus 8* (Muthén & Muthén, 1998–2018). MSEM estimates within- and between-person associations separately (Preacher et al., 2010). Within the multilevel models, we also included Add Health sampling weights to account for the study design and sampling (P. Chen & Harris, 2020). All models used maximum likelihood estimation with robust standard errors (MLR), which provides estimations of standard errors that are robust to nonnormal data (Yuan & Bentler, 2000). The intraclass correlation (ICC) showed that most of the variability in the endogenous variables was explained by individual characteristics. Specifically, the ICC values were as follows: 2 % for Wave IV depression, 3 % for Wave IV self-reported health, 3 % for Wave IV metabolic syndrome, and 13 % for Wave IV educational attainment. The current dataset included a small amount of missing data across key study constructs, with missingness ranging from 0 % to 17 %. Most covariates had minimal missing, except for Wave I GPA, which had 36 % missing. To address the missing data, we used full-information maximum likelihood (FIML), which estimates model parameters using all available data without imputing missing values (Yuan et al., 2012). Psychometric analyses showed that all the study variables were normally distributed, with skewness and kurtosis falling within acceptable ranges for normality (see Table 1).

We first examined the longitudinal associations between perceived teacher unfair treatment during adolescence and young adult health outcomes. At the individual level (within level), we included both perceived teacher unfair treatment and individual-level covariates. At the same time, at the school level (between level), we included school-wide perceptions of teacher unfair treatment along with school-level covariates. To account for variations in school-level perceptions, we centered the school-wide perceived teacher unfairness by subtracting the overall mean (i.e., the average across all schools) from each school's average score. The three health outcomes were analyzed simultaneously in the same model. In a separate model, we examined possible implications of discrepancies between adolescent and school-wide perceptions of teacher unfairness on the three health outcomes. In this model, the individual-school discrepancy score was included at the individual level, with individual- and school-level covariates controlled for at their respective levels.

In the next set of analyses, we examined whether educational attainment served as a potential individual-level mediator in the linking perceptions of teacher unfairness (at both the individual and school-levels) using the multilevel structural equation modeling framework. We estimated both direct and indirect effects. Indirect effect confidence intervals were estimated using Monte Carlo simulations of the respective effect sampling distributions, using R software (codes available at http://www.quantpsy.org/medmc/medmc.htm; Preacher et al., 2010). Across all the analyses, we controlled for individual- and school-level covariates, and each respective health outcome at Wave I (e.g., depressive symptoms at Wave 4 regressed on depressive symptoms at Wave I). Because metabolic syndrome indicators were not available at Wave I, we adjusted for Wave I BMI in the metabolic syndrome model.

Finally, we conducted supplementary analyses to examine whether perceptions of teacher unfairness varied across key demographic variables (i.e., race/ethnicity, gender, and parental education as a proxy for family socioeconomic status). These analyses were conducted using ANOVA in R software, with sampling weights applied to account for the study design.

## 3 Results

### 3.1 Individual-level and school-level direct effects

We first examined whether adolescents’ own perceptions of teacher unfair treatment, as well as school-wide perceptions, were linked to their health outcomes in young adulthood (i.e., depression, self-reported health, and metabolic syndrome). As shown in Table 4, after accounting for individual and school characteristics, as well as corresponding health outcomes at Wave I, higher individual-level perceptions of unfair treatment by teachers were significantly associated with increased depression at Wave IV (*b* = 0.14, *p* < .001). While higher individual-level perceptions of teacher unfair treatment showed a marginal association with poorer self-reported health (*b* = −0.02, *p* = .06), higher school-level perceptions were significantly associated with lower self-reported health at Wave IV (*b* = −0.15, *p* = .01). No significant direct effect on metabolic syndrome at Wave IV was found at either the individual level (*b* = −0.01, *p* = .58) or the school-level (*b* = 0.05, *p* = .41).

**Table 4:** Relationships between perceived teacher unfair treatment and health outcomes.

	W4 Depression		W4 Self-Reported Health	W4 Metabolic Syndrome		
	*b (SE)*	*p*	*b (SE)*	*p*	*b (SE)*	*p*
Individual-level Perceived Teacher Unfairness at W1	**0.14 (0.05)**	**<0.001**	−0.02 (0.01)	0.060	−0.01 (0.01)	0.580
School-Wide Perceived Teacher Unfairness at W1	0.32 (0.27)	0.240	**−0.15 (0.05)**	**0.010**	0.05 (0.07)	0.410
**Individual-level Covariates**						
Black	0.41 (0.16)	0.010	−0.03 (0.04)	0.470	0.12 (0.05)	0.010
Latinx	0.03 (0.18)	0.850	−0.1 (0.04)	0.010	0.07 (0.05)	0.140
Asian	0.48 (0.28)	0.090	−0.22 (0.08)	0.010	0.13 (0.11)	0.220
Other	0.42 (0.22)	0.050	−0.11 (0.05)	0.020	0.1 (0.07)	0.150
Gender (Male = 1, female = 0)	−0.47 (0.08)	<0.001	0.04 (0.02)	0.050	0.32 (0.03)	<0.001
Age at W1	−0.06 (0.04)	0.090	0 (0.01)	0.720	0.01 (0.01)	0.460
Parental Education (1 = With Postsecondary Degree)	−0.32 (0.09)	<0.001	0.1 (0.02)	<0.001	−0.13 (0.03)	<0.001
GPA at W1	−0.37 (0.1)	<0.001	0.11 (0.02)	<0.001	−0.12 (0.03)	<0.001
Household Income at W1	−0.1 (0.02)	<0.001	0.02 (0)	<0.001	−0.01 (0.01)	0.030
Depression at Wave I	0.26 (0.01)	<0.001	–	–	–	–
Self-reported Health at Wave I	–	–	0.25 (0.01)	<0.001	–	–
BMI at Wave I	–	–	–	–	0.09 (0)	<0.001
**School-level Covariates**						
Percent of Minority Students	0.00 (0.00)	0.550	0.00 (0.00)	0.250	0.00 (0.00)	0.380
Percent of New Teacher	0.00 (0.00)	0.860	0.00 (0.00)	0.250	0.00 (0.00)	<0.001
Percent of Teachers Who Worked at the Same School for Five Years or More	0.00 (0.00)	0.420	0.00 (0.00)	0.010	0.00 (0.00)	0.010
School Size	0.05 (0.08)	0.530	0.00 (0.02)	0.950	−0.02 (0.02)	0.380
Percent of Parents with Postsecondary Degree	0.32 (0.37)	0.400	0.01 (0.08)	0.860	−0.32 (0.12)	0.010
Percent of Households Receiving Welfare	2 (0.93)	0.030	−0.5 (0.18)	0.010	−0.43 (0.29)	0.130

*Note*. WI = Wave I, W4 = Wave IV; Significant results were in bold. White is the reference group for race.

### 3.2 Individual-school discrepancy direct effect

Next, we examined whether discrepancies between student and school-wide perceptions of teacher unfair treatment were related to health outcomes in young adulthood. When students perceived more unfair treatment from teachers than their peers did, they experienced significantly higher levels of depressive symptoms (*b* = 0.14, *p* = .004) and marginally lower self-reported health (*b* = −0.02, *p* = .052) in young adulthood, controlling for individual and school characteristics and the corresponding health outcomes from Wave I (Table 5). No significant results were observed for metabolic syndrome (*b* = −0.01, *p* = .71).

**Table 5:** Relationships between W1 individual-school discrepancy score and W4 health outcomes.

	W4 Depression		W4 Self-Reported Health		W4 Metabolic Syndrome	
	*b (SE)*	*p*	*b (SE)*	*p*	*b (SE)*	*p*
Individual-School Discrepancy Score at W1	**0.14 (0.05)**	**0.004**	−0.02 (0.01)	0.052	−0.01 (0.01)	0.708
**Individual-level Covariates**						
Black	0.41 (0.16)	0.012	−0.03 (0.04)	0.442	0.12 (0.05)	0.012
Latinx	0.03 (0.18)	0.89	−0.1 (0.04)	0.014	0.07 (0.05)	0.149
Asian	0.48 (0.28)	0.087	−0.22 (0.08)	0.007	0.13 (0.11)	0.217
Other	0.42 (0.22)	0.052	−0.11 (0.05)	0.023	0.1 (0.07)	0.154
Gender (Male = 1, female = 0)	−0.47 (0.08)	<0.001	0.04 (0.02)	0.049	0.32 (0.03)	<0.001
Age at W1	−0.06 (0.04)	0.104	0 (0.01)	0.531	0.01 (0.01)	0.424
Parental Education (1 = With Postsecondary Degree)	−0.32 (0.09)	<0.001	0.1 (0.02)	<0.001	−0.13 (0.03)	<0.001
GPA at WI	−0.38 (0.1)	<0.001	0.11 (0.02)	<0.001	−0.12 (0.03)	<0.001
Household Income at W1	−0.1 (0.02)	<0.001	0.02 (0)	<0.001	−0.01 (0.01)	0.031
Depression at Wave 1	0.26 (0.01)	<0.001	–	–	–	–
Self-reported Health at Wave 1	–	–	0.25 (0.01)	<0.001	–	–
BMI at Wave 1	–	–	–	–	0.09 (0)	<0.001
**School-level Covariates**						
Percent of Minority Students	0.00 (0.00)	0.682	0.00 (0.00)	0.42	0.00 (0.00)	0.343
Percent of New Teacher	0.00 (0.00)	0.851	0.00 (0.00)	0.258	0.00 (0.00)	<0.001
Percent of Teachers Who Worked at the Same School for Five Years or More	0.00 (0.00)	0.433	0.00 (0.00)	0.017	0.00 (0.00)	0.011
School Size	0.09 (0.08)	0.251	−0.01 (0.02)	0.393	−0.02 (0.02)	0.472
Percent of Parents with Postsecondary Degree	0.23 (0.39)	0.547	0.05 (0.09)	0.601	−0.33 (0.12)	0.004
Percent of Households Receiving Welfare	1.99 (0.97)	0.039	−0.51 (0.19)	0.008	−0.43 (0.29)	0.135

*Note*. WI = Wave I, W4 = Wave IV. Significant results were in bold. White is the reference group for race.

### 3.3 Individual-level and school-level mediation effects

**Fig. 1:**
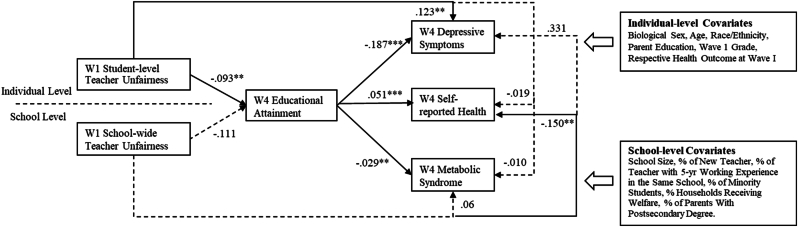
Multilevel mediation models linking teacher unfairness (at both indiviudal- and school-levels), Wave IV educational attainment, and Wave IV health outcomes. W1 = Wave I, W4 = Wave IV. Significant paths were shown in solid line and non-significant paths were shown in dashed line. ∗*p* < .05, ∗∗*p* < .01, ∗∗∗*p* < .001.

Individual perceptions of teacher unfairness were indirectly linked to all three health outcomes at Wave IV through educational attainment, based on estimated indirect effects from mediation analyses (Table 6). Perceiving higher levels of unfair treatment by teachers at the individual level in Wave I was associated with lower educational attainment (*b* = −0.09, *p* = .001) by Wave IV (Fig. 1). This lower educational attainment, in turn, was linked to higher levels of depressive symptoms (*b* = −0.19, *p* < .001), poorer self-rated health (*b* = 0.05, *p* < .001), and higher levels of metabolic syndrome (*b* = −0.03, *p* = .001) at Wave IV. No significant mediating effects were found for school-level perceptions of teacher unfairness.

**Table 6:** Estimates of indirect effects for educational attainment for the longitudinal link between teacher unfairness and adult health.

	Indirect effect		Percent mediated
	*b*	95 % CI	
**Individual-level Teacher Unfairness (W1) - > Educational Attainment (W4) - > Depressive Symptoms (W4)**	**0.017**	**[0.007, 0.027]**	**13 %**
School-Wide Teacher Unfairness (W1) - > Educational Attainment (W4) - > Depressive Symptoms (W4)	0.021	[-0.04, 0.081]	–
**Individual-level Teacher Unfairness (W1) - > Educational Attainment (W4) - > Self-Reported Health (W4)**	**−0.005**	**[-0.007, -0.002]**	**26 %**
School-Wide Teacher Unfairness (W1) - > Educational Attainment (W4) - > Self-Reported Health (W4)	−0.006	[-0.022, 0.011]	–
**Individual-level Teacher Unfairness (W1) - > Educational Attainment (W4) - > Metabolic Syndrome (W4)**	**0.003**	**[0.001, 0.005]**	**30 %**
School-Wide Teacher Unfairness (W1) - > Educational Attainment (W4) - > Metabolic Syndrome (W4)	0.003	[-0.006, 0.013]	–

*Note.* Significant pathways were in bold. W1 = Wave I. W4 = Wave IV.

### 3.4 Supplementary analyses

Supplementary analyses revealed significant differences in perceptions of teacher unfairness across race/ethnicity (*F* (4, 9905) = 6.80, *p* < .001), gender (*F* (1, 9910) = 4.17, *p* = .04), and family socioeconomic status (*F* (1, 9912) = 18.53, *p* < .001). Post hoc analyses indicated that Black respondents reported significantly higher levels of teacher unfairness compared to White, Asian, and Latino respondents. Additionally, girls reported higher levels of teacher unfairness than boys, and individuals whose parents had completed a postsecondary degree reported lower levels of teacher unfairness compared to those whose parents had not.

## 4 Discussion

Guided by the social determinant of health framework (Viner et al., 2012), this study is among the first to document the long-lasting health consequences of perceived teacher unfairness during adolescence at multiple levels (individual, school-wide, and individual-school discrepancy). Our results underscore the negative effects of perceived teacher unfair treatment during adolescence on mental and physical health in young adulthood, with particularly pronounced consequences when an adolescent's perceptions of teacher unfairness exceed those of their peers. In addition, results from our mediation analyses support reduced educational attainment as a mechanism explaining the harmful effect of teacher unfairness on later negative health outcomes.

The first goal of the study was to examine the long-term impact of adolescents’ perceptions of teacher unfairness and their health outcomes 13 years later in young adulthood. Our finding reveals that adolescents who perceived greater unfair treatment by their teachers during adolescence reported significantly higher levels of depressive symptoms and a trend of poorer self-reported health in adulthood, even after controlling for a wide variety of potentially confounding individual- and school-level covariates. This finding aligns with *equity and social exchange theory* (Adams, 1965), which posits that perceived inequity can evoke negative emotions and contribute to physical distress (Respress et al., 2013). Our finding builds on prior research demonstrating the short-term effects of teacher unfairness on students' psychological well-being and somatic problems (F. Chen & Cui, 2020; Gini et al., 2018; Huang, 2020; Lenzi et al., 2013), by highlighting the enduring and far-reaching consequences of these experiences, which persist into adulthood. Our results underscore the urgency of fostering an inclusive and equitable social climate within schools, where all students can expect fair treatment. This is particularly crucial during adolescence, as addressing exposure to inequitable school climates during this formative period can reduce lifelong health risks and population-level health disparities (Viner et al., 2012). Educational policymakers should prioritize anti-bias training for teachers and implement regular assessments of school climate to identify disparities in perceived fairness. Public health systems must collaborate with schools to provide resources and support for at-risk youth, addressing inequities that contribute to long-term disparities in health. Cross-sector partnerships can play a pivotal role in designing and scaling interventions, ensuring that schools serve as equitable environments that promote both educational and health equity.

Furthermore, our results reveal an important trend: even after accounting for individual perceptions, the overall level of perceived teacher unfairness at school remained significantly linked to poorer self-reported health in young adulthood. Specifically, students who attend schools where the average level of perceived teacher unfairness was high reported worse physical health 13 years later. This finding underscores the impact of adverse school environments, which may reflect broader systemic issues within the institution. The weathering theory of health disparities suggests that health outcomes are influenced by social, physical, and economic environmental conditions (Geronimus et al., 2020). Persistent stress from unfair teacher treatment within institutional settings can lead to physiological dysregulation, making individuals more susceptible to health problems over time and potentially accelerating the onset of health issues (Geronimus et al., 2006).

The finding that school-level teacher unfairness was associated with self-reported health but not depressive symptoms may be attributed to the distinct nature of these outcomes and how they are shaped by different levels of exposure. Specifically, self-reported health is a broad, subjective assessment of overall well-being (Wuorela et al., 2020) and is therefore more sensitive to environmental stressors, including systemic unfairness within the school climate. In contrast, depression is a more specific psychological construct, often driven by personal experiences (Orphanidou et al., 2023), which may explain its stronger association with individual-level teacher unfairness. These findings underscore the importance of examining phenomena at both individual and contextual levels, as different levels of analysis may reveal distinct pathways through which environmental stressors impact health and well-being.

Interestingly, neither individual-level nor school-wide perceptions of teacher unfairness were significantly associated with metabolic syndrome. It is possible that there may be moderating factors that are contributing to these null findings. For example, one study found that, for individuals with low maternal nurturance, childhood disadvantage was associated with higher counts of metabolic syndrome; however, this association was not significant for those with high maternal nurturance (Miller et al., 2011). Moving forward, future studies should continue exploring the moderating factors (e.g., parenting, psychological resilience, coping strategies) to gain a more comprehensive understanding of the factors underlying the link between teacher unfairness and metabolic syndrome.

In addition to the health consequences of individual and collective perceptions of teacher unfairness in schools, we also examined whether discrepancies between adolescents’ perceptions of teacher unfairness and those of their peers during adolescence would be particularly detrimental to their health outcomes in young adulthood. This approach acknowledges a complex interplay between individual and school characteristics, in line with the person-process-context framework (Bronfenbrenner & Morris, 2006). Consistent with our hypothesis, adolescents who perceived higher levels of teacher unfairness compared to their peers, reported significantly increased depressive symptoms and showed a trend of poorer self-reported health in young adulthood. This suggests that when adolescents perceive more teacher unfair treatment than their schoolmates, the adverse effects of perceived teacher unfairness are more pronounced. In such cases, adolescents may feel a heightened sense of isolation, making it harder to dismiss or rationalize their perceptions of unfairness. These findings align with previous research that highlights the heightened impact of victimization and peer prejudice on adolescent development, especially when such mistreatment is not a common experience in the school environment (Bellmore et al., 2004; Benner et al., 2015).

To address the question of how adolescent perceptions of teacher unfairness contribute to young adult health, we examined the mediating role of educational attainment. As hypothesized, adolescents who perceived unfair teacher treatment were more likely to experience lower educational achievement, which was in turn linked with increased depressive symptoms, poorer self-reported health, and higher metabolic syndrome risk in young adulthood. Prior research has shown that perceived teacher unfairness discourages student engagement, diminishes motivation, and elevates schoolwork-related anxiety, ultimately resulting in lower academic performance, higher school dropouts, and lower educational attainment (F. Chen & Cui, 2020; Helm et al., 2020; Huang, 2020; Lee & Breen, 2007; Ripski & Gregory, 2009). As educational attainment is a key determinant of health (Montez & Friedman, 2015), lower educational attainment can limit access to fulfilling careers, economic stability, and healthy lifestyles (Mirowsky & Ross, 2003), resulting in poorer mental and physical health in adulthood. Our findings underscore the importance of investing in educational resources to help mitigate the negative effects of perceived teacher unfairness. For instance, Chen and Cui (2020) found that schools with greater resources can mitigate the negative impact of perceived teacher unfairness on students’ academic achievement. In these schools, students benefit from a broader range of institutional resources that protect them when they encounter unfair teacher treatment. Consequently, intervention programs that strengthen the capacity of teachers and school counselors to provide academic assistance and mentorship are crucial for mitigating the negative effects of teacher unfairness. Such support can help motivate students who may grapple with teacher unfairness, keeping them engaged in school and reducing the risk of dropout.

Interestingly, educational attainment mediated a larger proportion of the total effect of teacher unfairness on self-reported health (26 %) and metabolic syndrome (30 %) compared to depressive symptoms (13 %). This finding suggests that education, by facilitating access to resources and healthcare (Mirowsky & Ross, 2003), may exert a stronger influence on physical health outcomes. In contrast, the link between teacher unfairness and depression may also be mediated by psychological factors, such as self-esteem and self-efficacy, which are closely tied to depressive symptoms (Hermann & Betz, 2006). These findings highlight the need for future research to explore alternative mediators that may better explain the pathways between unfairness and mental health outcomes. Understanding these mechanisms could inform the development of targeted interventions to mitigate the negative effects of teacher unfairness on psychological well-being.

It is worth noting that educational attainment did not mediate the link between school-wide perceptions of teacher unfairness during adolescence and self-reported health in young adulthood. This suggests that there might be additional, unexplored pathways at play. It is possible that, in adverse and unfair environments, students may feel compelled to work harder and strive to please their teachers to avoid becoming targets of unfair treatment. This heightened effort might help protect their educational attainment, but the exertion under social stress can contribute to heightened biological stress, negatively impacting physical health. This idea aligns with the John Henrysim phenomenon (Bennett et al., 2004), which describes how individuals from marginalized backgrounds, when faced with social stress, often rely on traits such as self-discipline and perseverance to achieve outward success—at the cost of their physical health due to the chronic activation of the stress-response system. There is still much to uncover about the mechanisms through which school-level perceptions of teacher unfairness influence individual health outcomes. Therefore, we encourage future research to explore these complex pathways in greater depth.

Supplementary analyses revealed significant disparities in perceptions of teacher unfairness across race, gender, and socioeconomic status, reflecting systemic inequities in education (Jones et al., 2020). Black students reported significantly higher levels of perceived unfairness compared to their White, Asian, and Latino peers, underscoring the racialized nature of teacher bias and aligning with research showing that such biases disproportionately impact racial/ethnic minority students (Owens, 2022). These biases not only affect educational experiences but may also perpetuate race- and SES-based health disparities through chronic stress and adverse psychosocial outcomes. Gender differences were also observed, with girls reporting higher levels of perceived unfairness than boys, possibly due to greater scrutiny or harsher evaluations in certain contexts, particularly in male-dominated STEM subjects (Handley et al., 2015). Additionally, socioeconomic status differences revealed that students whose parents had completed postsecondary education reported lower levels of teacher unfairness compared to those whose parents had not, suggesting that implicit class-based biases and systemic barriers play a role in shaping these perceptions (Sneyers et al., 2020). Addressing these disparities calls for targeted interventions, including culturally responsive and gender-equitable teaching practices as well as systemic reforms to promote fairness and equity in schools, especially among marginalized student groups. Additionally, population-level policies are also crucial to reduce systemic inequities in school funding and resources, which disproportionately impact low-income and minority students.

### 4.1 Strengths, limitations, and future directions

The current study makes several key contributions. First, by using a 13-year large prospective national sample, we demonstrated the long-term negative effects of perceived teacher unfairness during adolescence on young adult health. Our findings provide compelling evidence that perceptions of unfair treatment during adolescence can have lasting impacts on mental and physical health in young adulthood. These results emphasize the importance of early-life social experience and their potential to shape long-term health disparities, highlighting adolescence as a critical developmental stage during which such experiences become "socially embedded.” Second, we took an innovative approach by examining a phenomenon typically analyzed at the individual level with the broader context of school-level climate. Our research shows that schoolmates’ collective perceptions of teacher unfairness offer a particularly valuable measure of school climate and generalized teacher behavior, warranting greater attention in future studies. Third, the current study integrates both mental and physical health indicators, using a combination of self-reported and objective measures. This comprehensive approach strengthens the robustness of our findings, as many studies tend to focus on only one type of outcome or rely solely on self-report data.

In addition to the novel and significant contributions of this study, limitations must be acknowledged. First, this study is correlational in nature. Although we used longitudinal data and accounted for key individual and school-level covariates, we cannot establish causal relationships. Second, the Add Health data includes only a single item related to perceptions of teacher unfairness. This item does not distinguish between adolescents being direct victims of or witnesses to teacher unfair treatment. Future research should employ a more comprehensive operationalization that differentiates between personal and vicarious experiences of teacher unfair treatment to deepen our understanding of these dynamics. Third, the assessment of teacher unfair treatment in Add Health is relatively broad. Drawing from the classroom justice literature (Chory‐Assad & Paulsel, 2004; Molinari et al., 2013), there are typically three distinct dimensions to consider: distributive justice (fairness in grading compared to a standard rule), procedural justice (fairness in the procedures used by teachers for making decisions related to classroom outcomes), and interactional justice (fairness on how teachers interact with students regarding their communication and relational requests). Future studies should use more nuanced measures of teacher unfairness to explore how each of these dimensions uniquely impacts long-term health outcomes. Finally, when examining discrepancies between students' and their peers’ perceptions of teacher unfairness, we calculated the difference scores. Although commonly used, this method has limitations, such as providing less clarity about the absolute magnitude of the differences (Hou et al., 2020). To address this, future research could employ alternative methods, like polynomial regression and response surface analyses (Barranti et al., 2017) for deeper insights into how discrepancies in perceptions relate to later health outcomes.

## 5 Conclusions

Using a 13-year prospective longitudinal national sample, this study uncovered an enduring association between perceptions of teacher unfairness experienced in adolescence and mental as well as physical health in young adulthood. Our findings underscore the pivotal role schools need to play in promoting fairness and nurturing the well-being of future generations. They also highlight the need for educator training to minimize unfair treatment in classroom settings and to ensure equitable, inclusive learning environments. Fostering trust in teachers could also help promote student perceptions of fairness. Finally, our findings highlight that education attainment mediates the association between greater individual perceptions of teacher unfairness and later worse health outcomes. Overall, policies and interventions aimed at promoting teacher fairness, building trust in educators, and supporting students who experience teacher unfairness are essential for creating equitable school environments, reducing health disparities, and improving population-level outcomes in morbidity and mortality.

## CRediT authorship contribution statement

**Shanting Chen:** Writing – review & editing, Writing – original draft, Visualization, Validation, Project administration, Methodology, Investigation, Formal analysis, Conceptualization. **Stephanie Koning:** Writing – review & editing, Supervision, Conceptualization. **Jessica Polos:** Writing – review & editing. **Phoebe Lam:** Writing – review & editing. **Taylor Hargrove:** Writing – review & editing. **Natalie Ebner:** Writing – review & editing. **Jacob Aronoff:** Writing – review & editing. **Thomas McDade:** Writing – review & editing, Funding acquisition.

## Ethical statement

This study used data from the National Longitudinal Study of Adolescent Health (Add Health). This study was approved by the institutional review board of the University of North Carolina, Chapel Hill, and all participants provided written informed consent.

## Declaration of competing interest

The authors declare that they have no known competing financial interests or personal relationships that could have appeared to influence the work reported in this paper.

## Data Availability

The authors do not have permission to share data.
